# A dataset of assessing factors influencing online math-ICT integration among Indonesian preservice mathematics educators: A pilot study

**DOI:** 10.1016/j.dib.2024.111015

**Published:** 2024-10-10

**Authors:** Naufal Ishartono, Siti Hajar binti Halili, Rafiza binti Abdul Razak, Muhammad Noor Kholid

**Affiliations:** a Department of Mathematics Education, Faculty of Teacher Training and Education, Universitas Muhammadiyah Surakarta, 57169 Central Java, Indonesia; b Department of Curriculum and Instructional Technology, Faculty of Education, University of Malaya, 50603 Kuala Lumpur, Malaysia

**Keywords:** Math-TPACK, Beliefs on ICT, Beliefs on online learning, Theory of planned behavior, SEM-PLS, SPSS

## Abstract

This paper presents a dataset from a pilot study of a questionnaire designed to identify factors influencing Indonesian preservice mathematics teachers' integration of digital mathematics learning media (DMLM) during online teaching practice. The dataset was gathered through an online questionnaire administered from October to November 2022 in Indonesia, with 303 preservice mathematics teachers from 14 universities across 11 provinces voluntarily participating. The respondents were characterized by their online learning experience, ongoing teaching practice, and enrollment in mathematics education programs. The questionnaire addressed three main factors: Math-TPACK, Beliefs on Online Learning, and Beliefs on DMLM, and was adapted from previous studies measuring similar aspects. The instrument underwent face and content validity checks by involving experts. Additionally, the Partial Least Squares Structural Equation Model (PLS-SEM) was used to assess the validity and reliability of the measurement model. This statistical process examined the loadings of reflective indicators, internal consistency reliability, and both convergent and discriminant validity. This dataset is valuable for practitioners, researchers, and stakeholders in mathematics education for developing programs that support preservice mathematics teachers in integrating DMLM during online teaching practice.

Specifications Table

**Table:** 

Subject	Education
Specific subject area	Educational Technology
Type of data	Table Figure
Data collection	Instrument from previous studies were adapted based on literature review (see Table 2) and validated through content validity. Further, it was translated from English to Indonesian language. In the data preparation, the computation of skewness and Kurtosis were done. Reliability assessment was done through Cronbach's alpha. Exploratory Factor Analysis (EFA) were addressed for the three main constructs: Math-TPACK, Beliefs on Digital Mathematics Learning Media, and Beliefs on Online Learning.
Data source location	The data were collected from 14 universities in 11 Indonesian provinces
Data accessibility	Repository name: Pilot Data of Instrument Validation for Factor Affecting Indonesian Preservice Mathematics Teachers' Technology Integration Data identification number: 10.17632/vw2m9w8b2c.1 Direct URL to data: https://data.mendeley.com/datasets/vw2m9w8b2c/1 Instructions for accessing these data: Data terdiri dari raw data, instruments in Bahasa Indonesia and English, and the analysis result of SmartPLS 3 and SPSS 23

## 1 Value of the Data

• Valid and reliable datasets are required to support studies on Digital Mathematics Learning Media (DMLM) integration during the Indonesian preservice mathematics teachers' online teaching practice.
• The dataset provides factors affecting Indonesian preservice mathematics teachers' DMLM integration during online teaching practice.
• The dataset can be used by practitioners in mathematics education as a consideration in designing the best program to guide the Indonesian preservice mathematics teachers' DMLM integration during online teaching practice.
• Stakeholders in mathematics education can utilize the open-access dataset to understand the practices of DMLM in the preservice mathematics teacher training program.
• Researchers who study similar samples and variables in developing countries can adapt this dataset for more rigorous statistical analysis.

## 2 Background

Mathematics is a study about abstract objects such as geometric shapes, numbers, symbols, and logic [1]. In the context of mathematics education, the most significant aspect of a student's ability to understand mathematical concepts is their abstraction skills. Abstraction strengthens students' early knowledge by allowing them to recognize and generalize patterns and principles from individual examples. This technique allows pupils to create a strong foundation for more difficult mathematical ideas [2]. The problem that arises is that not all students have good abstraction skills [3]. Therefore, to bridge between mathematical concepts and students' understanding, a mathematics learning medium is needed either as a visualization tool, confirmation tool, or assessment tool [4]. Based on these conditions, a mathematics teacher is required to be agile and skilled in using mathematics learning media during mathematics learning process.

Since the Covid 19 pandemic began in early 2020, many countries, including Indonesia, have moved their learning processes online. In early March 2020, the Indonesian Ministry of Education issued a decree on the implementation of education during the Covid-19 emergency [5]. The directive has caused all Indonesian schools to transfer their learning from offline to online. The issue was that not all teachers prepared to carry out the online teaching and learning process, both in terms of their talents and the equipment they have [6], not to mention mathematics teachers. Indonesian mathematics teachers confront issues not only in administering online mathematics learning, but also in using digital mathematics learning media to depict mathematical concepts [7]. However, with the passage of time, the Indonesian mathematic in-service teachers received a lot of training from various institutions, both government and private, so that their skills became better in administering online mathematics learning [[8], [9], [10], [11]].

## 3 Data Description

The dataset contains the findings of a pilot research of a questionnaire designed to identify the factors impacting Indonesian preservice mathematics teachers' (PSMTs) use of digital mathematics learning media during online teaching practices. The dataset was obtained using a survey technique with a sampling technique, namely convenient random sampling. The participants in this study were Indonesian PSMTs from 14 universities in Indonesia and from 11 provinces. The characteristics of the participants were preservice mathematics teachers who had gained online learning experience, had or were currently undergoing teaching practice, and were in their minimum third year.

**Fig. 1:**
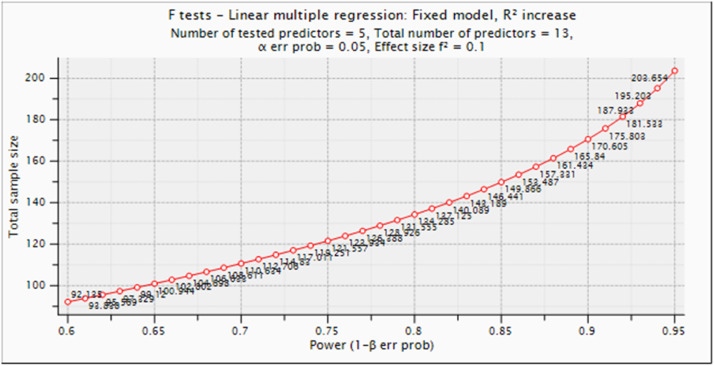
G*Power Result.

In this study, the determination of the number of participants was based on the survey’ power calculation by using G*Power application (see Fig 1). The G*Power was initially developed by [12], and a general stand-alone power analysis program for statistical tests that are commonly used in social and behavioral research was developed for that. This version of G* power, version 3.1.9.4, is a free application that is an improvement over the previous version. It is compatible with the most popular platforms for used laptops, such as Windows 10, and it features a variety of statistical procedures, including the *t*-test, the F test, and the O^2^ test families. In addition to that, it offers power analyses for z-tests in addition to some exact tests. In this study, the researcher used multiple linear regression with 13 predictors and five tested predictors. The f^2^ value used was 0.1, and there were 13 predictors in total. The application recommends collecting 203.7 or 204 samples as the minimum sample size.

The questionnaire is established on two theoretical frameworks: TPACK (Technological Pedagogical Content Knowledge) and the Theory of Planned Behavior (TPB). In TPACK, Schmidth defines TPACK as a helpful framework for thinking about what knowledge teachers must have to integrate TPACK as a framework for measuring teaching knowledge which potentially impact the type of training and professional development experiences designed for both preservice and in-service teachers [13]. As a result, Math-TPACK can be defined as a TPACK framework based on mathematical knowledge and software. The TPACK framework is made up of three types of knowledge: technological knowledge (TK), pedagogical knowledge (PK), and content knowledge (CK). Furthermore, TPACK is a combination of three basic knowledge: pedagogical-content knowledge (PCK), technological-content knowledge (TCK), technological-pedagogical-pedagogical-content knowledge (TPK), and technological-pedagogical-content knowledge (TPACK). In this study, TPACK was modified into Math-TPACK or TPACK which is based on mathematical aspects in each component.

**Fig. 2:**
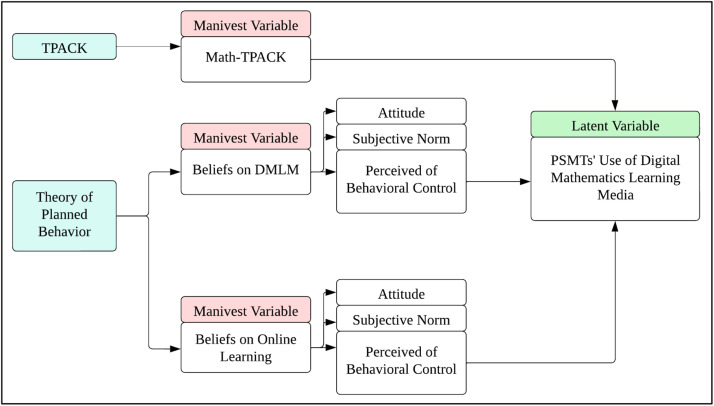
Conceptual Framework.

The next is Theory of Planned Behavior that introduced by Ajzen in 1980 as an academic framework aimed at understanding someone's belief in things, objects, or ways [14]. In the questionnaire, TPB is used as the basis of two factors, namely Beliefs on Digital Mathematics Learning Media (DMLM) and Beliefs on Online Learning (OL). Both factors are based on the Theory of Planned Behavior framework developed by Habibi [15] which is based on three components, namely Attitude (AT), Subjective Norm (SN), and Perceived of Behavioral Control (PBC). Attitude (AT) is a person's overall evaluation or positive/negative feeling toward performing a specific behavior. It entails a person's beliefs and perceptions about the likely outcomes or consequences of that behavior. Personal values, beliefs, experiences, and social influences all contribute to the formation of attitudes. Positive attitudes toward a behavior are more likely to result in a favorable intention to engage in that behavior, whereas negative attitudes are more likely to discourage individuals from engaging in it [14]. While Subjective Norm (SN) refers to an individual's perception of social pressure or influence from important others (e.g., friends, family, colleagues, or society) regarding the performance or non-performance of a specific behavior [14]. Lastly, Perceived Behavioral Control (PBC) refers to an individualʼs perception of their ability to successfully perform a behavior [14]. Based on this explanation, the Beliefs on DMLM and Beliefs on OL factors are composed of these three components. The Beliefs on DMLM factor consists of the components Attitude toward DMLM (DMLM-AT), Subjective Norm toward DMLM (DMLM-SN), and Perceived Behavioral Control toward DMLM (DMLM-PBC). While the Beliefs on OL factor is also composed of components of Attitude toward OL (OL-AT), Subjective Norm toward OL (OL-SN), and Perceived Behavioral Control toward OL (OL-PBC). Overall, the three factors are summarized in the conceptual framework which can be seen in Fig 2.

In the construction aspect, the questionnaire is developed based on 5-point Likert's Scale: 1 (Strongly Disagree), 2 (Disagree), 3 (Between Disagree or Agree), 4 (Agree), and 5 (Strongly Agree) [16]. In addition, the questionnaire consists of two parts, namely demographic information, and main survey (Math-TPACK, Beliefs on DMLM, and Beliefs on OL). In the demographic information section, there are three questions addressed to students, namely related to gender (male and female), DMLM-based course experienced by the students (≤ 2 courses and > 2 courses), university background (A-Accredited and Non-A Accredited). The analysis result of the demographic information can be seen in Table 1. While in the main survey section, which consists of thirteen constructions and 63 questions (see Table 4).

**Table 1:** Demographic information (n. 303).

Demographic	n	%
**Gender** Male Female	81 221	27 73
**DMLM Based Course** ≤ 2 courses > 2 courses	161 142	53 47
**University Accreditation** A-Accredited Non-A Accredited	249 54	82 18

The questionnaire was then tested for internal validity using item content validity index (I-CVI) and scale content validity index (S-CVI) based on assessments by seven experts in the field of education from two universities in Malaysia and three universities in Indonesia. The experts’ criteria are those who hold a doctorate degree in education and have had quantitative research experience as well as publications in international journals indexed by Scopus for at least the last three years. Table 2 shows the results of the content validity analysis for a questionnaire designed to evaluate various aspects, including Math-TPACK, Beliefs on Digital Mathematics Learning Media (DMLM), Beliefs on Online Learning (OL), and Use of Digital Mathematics Learning Media (UDMLM). Each aspect is assessed through specific items: Quality Indicator (QI), Fluency (FL), and Ease of Use (EU). For each item, six experts provided ratings, resulting in an Item Content Validity Index (I-CVI) of 1 for all items, indicating unanimous agreement on their relevance. The Universal Agreement (UA) column shows a score of 1, signifying that all experts fully agreed on the relevance of every item. Consequently, the Scale Content Validity Index Average (S-CVI/Ave) and the Scale Content Validity Index Universal Agreement (S-CVI/UA) for each aspect are also 1, highlighting the consistent and high validity of the questionnaire items across all evaluated dimensions. Overall, the findings suggest that the questionnaire items were unanimously deemed relevant by the experts, ensuring high content validity. Each aspect—Math-TPACK, Beliefs on DMLM, Beliefs on OL, and UDMLM—achieved perfect scores in both individual item validity (I-CVI) and universal agreement (UA). This comprehensive validation process underscores the robustness of the instrument in accurately measuring the intended factors, making it a reliable tool for assessing the integration of digital mathematics learning media among Indonesian preservice mathematics teachers during online teaching practices.

**Table 2:** Content Validity Index (CVI) Result.

Aspect	AA	EA	I-CVI	I-CVI Category	UA	S-CVI/Ave	S-CVI/UA
Math-TPACK	QI	6	1	Relevant	1	1	1
	FL	6	1	Relevant	1		
	EU	6	1	Relevant	1		
Beliefs on DMLM	QI	6	1	Relevant	1	1	1
	FL	6	1	Relevant	1		
	EU	6	1	Relevant	1		
Beliefs on OL	QI	6	1	Relevant	1	1	1
	FL	6	1	Relevant	1		
	EU	6	1	Relevant	1		
UDMLM	QI	6	1	Relevant	1	1	1
	FL	6	1	Relevant	1		
	EU	6	1	Relevant	1		

Note: AA = Assessed Aspect; EA = Expert in Agreement; Ave = Average; UA = Universal Agreement; QI = The statement follows the questionnaire indicators; FL = Statement using the formal language; EU = Statements are seen as easy to understand by respondents.

Furthermore, Table 3 provides details the evolution of a questionnaire used to assess various domains related to the integration of digital mathematics learning media (DMLM) by Indonesian preservice mathematics teachers. It compares three versions of the questionnaire (Version 1a, Version 2b, and Version 3c), showing the number of indicators for each dimension within the domains of Math-TPACK, Beliefs on DMLM, Beliefs on Online Learning (OL), and Use of DMLM (UDMLM). Each dimension within these domains, such as Technological Knowledge (TK), Content Knowledge (CK), Pedagogical Knowledge (PK), and others, has a specified number of indicators that have been adjusted over the different versions of the questionnaire. In the Math-TPACK domain, the indicators for Technological Knowledge (TK) were reduced from 5 in Version 1a to 3 in Versions 2b and 3c, reflecting a refinement process likely aimed at increasing the focus and precision of the measurement. Other dimensions within Math-TPACK, such as Content Knowledge (CK), Pedagogical Knowledge (PK), and Technological Pedagogical Content Knowledge (TPACK), maintained a consistent number of indicators across all versions, suggesting that these dimensions were considered stable and comprehensive from the start. This consistency indicates that while some aspects of the questionnaire were streamlined, the core constructs remained robust. For the domains Beliefs on DMLM and Beliefs on OL, the number of indicators for Attitude (AT), Subjective Norms (SN), and Perceived Behavioral Control (PBC) remained consistent across all three versions, signifying a stable measurement framework for these beliefs. The Use of DMLM domain saw a slight reduction in indicators from 7 in Version 1a to 6 in Versions 2b and 3c. This adjustment reflects an effort to optimize the questionnaire's effectiveness and efficiency. Overall, the total number of indicators decreased from 68 in Version 1a to 64 in Versions 2b and 3c, indicating a refinement process aimed at streamlining the instrument while maintaining its comprehensiveness and validity.

**Table 3:** The different versions of the domains during the validation.

Domains	Version 1^a^		Version 2^b^ (64 indicators)		Version 3^c^ (64 indicators)	
	Dimension	Number of Indicators	Dimension	Number of Indicators	Dimension	Number of Indicators
Math-TPACK	TK	5	TK	3	TK	3
	CK	4	CK	4	CK	4
	PK	6	PK	6	PK	6
	PCK	5	PCK	5	PCK	5
	TCK	5	TCK	4	TCK	4
	TPK	4	TPK	4	TPK	4
	TPACK	5	TPACK	5	TPACK	5
Beliefs on DMLM	AT	5	AT	5	AT	5
	SN	4	SN	4	SN	4
	PBC	4	PBC	4	PBC	4
Beliefs on OL	AT	5	AT	5	AT	5
	SN	4	SN	4	SN	4
	PBC	5	PBC	5	PBC	5
UDMLM	–	7	–	6	–	6

**Total**		**68**		**64**		**64**

a Before the expert evaluation because of phase 1.

b Phase 2 (discussion with six experts).

c Phase 3 (discussion with five users).

After being declared to have passed the internal validity test using CVI tests, the authors got a professional translator who has doctoral degree on education to translate the questionnaire from English to Indonesian Language. Afterward, the questionnaire was tested for external validity through a pilot test. The sample used in the pilot test is based on the results of the G*Power calculation, which is above 204 respondents as shown in Fig 1.

The pilot study was conducted from October to November 2022. By using convenience random sampling technique, the author distributed the online questionnaires to 350 preservice mathematics teachers (PSMTs) from 14 Indonesian universities spread across 11 provinces. The universities were chosen based on the availability of Department of Mathematics Education in each university. The respondents voluntarily participated to fill the questionnaire and declare it in the self-declaration section embedded in the questionnaire form. Of the 350, 325 respondents filled out questionnaires. Then the data was analyzed using SPSS 23 to find and eliminate outliers where 22 data were eliminated and left as many as 303 data to be analyzed. The response rate received and can be analyzed is 86.5 %.

**Fig. 3:**
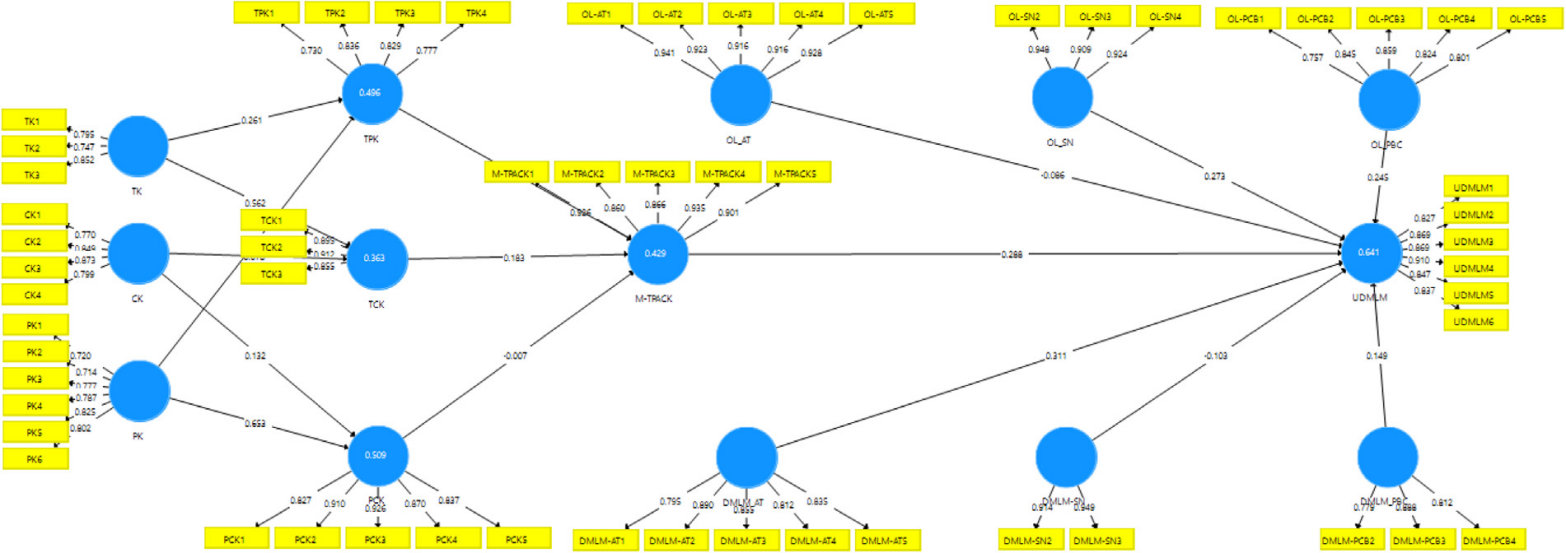
Measurement model.

The pilot study data was then statistically analyzed using two software, namely SPSS 23 and SmartPLS 3. SPSS 23 software is used to perform descriptive statistical analysis consisting of mean, SD, kurtosis, and skewness (see Table 4), and perform exploratory factor analysis (EFA). Then SmartPLS 3 software is used to determine reflective indicator loadings, internal consistency, composite reliability, and convergent validity [25] (Table 5). This software is chosen since it allows to model formative constructs, which are constructs formed by their indicators rather than determining them. Next is to determine the Forner-Larcker criteria for evaluating the validity of the questionnaire construct (Table 6). In addition, the software is used to determine the value of cross loading which shows a significant correlation between questions (Table 6). Finally, the software is also used to visualize the measurement model (Fig. 3).

**Table 4:** Variables' dimension, definition, and adapted references of the survey instruments.

Variable	Dimension	Definition	Components	Items (n)	Adapted references of the survey instruments
Math-TPACK	Technological Knowledge (TK)	Knowledge of emerging technologies for DMLM integration during teaching practice	TK1, TK2, TK3	3	[17]
	Content Knowledge (CK)	Knowledge of mathematical concepts and matter	CK1, CK2, CK3, CK4	4	[18,19]
	Pedagogical Knowledge (PK)	Knowledge of teaching, such as teaching principles, students' psychology of students, teaching strategies, and management of class during teaching practices	PK1, PK2, PK3, PK4, PK5, PK6	6	[17]
	Pedagogical and Content Knowledge (PCK)	Knowledge of transforming mathematics concepts into an understandable and accessible form for learners through a mathematics pedagogical approach during teaching practices.	PCK1, PCK2, PCK3, PCK4, PCK5	5	[17]
	Technological and Content Knowledge (TCK)	Knowledge of how to integrate DMLM for specific subject matter knowledge that excludes pedagogical goals during teaching practices.	TCK1, TCK2, TCK3, TCK4	4	[17]
	Technological and Pedagogical Knowledge (TPK)	Understanding of how to incorporate DMLM into pedagogy during teaching practices	TPK1, TPK2, TPK3, TPK4	4	[18,19]
	Mathematics Technological Pedagogical Content Knowledge (MTPACK)	Comprehension of how to use DMLM to increase students' understanding and acquisition of mathematical concepts during instructional activities	MTPACK1, MTPACK2, MTPACK3, MTPACK4, MTPACK5	5	[[17], [18], [19]]
Beliefs on DMLM	Attitude on DMLM (AT DMLM)	Associated with the perspective of the PSMTs in the use of DMLM during teaching practice	DMLM-AT1, DMLM-AT2, DMLM-AT3, DMLM-AT4, DMLM-AT5	5	[15,20,21]
	Subjective norm on DMLM (SN DMLM)	Associated with support system of the PSMTs environment in integrating the DMLM during teaching practice	DMLM-SN1, DMLM-SN2, DMLM-SN3, DMLM-SN4	4	[15,22]
	Perceived on Behavioral Control on DMLM (PBC DMLM)	Associated with internal and external enablers/constraints in using DMLM during teaching practice	DMLM-PBC1, DMLM-PBC2, DMLM-PBC3	3	[15,20,21]
Beliefs on Online Learning (OL)	Attitude on OL (AT OL)	Associated with the perspective of the PSMTs in the use of online learning mode during teaching practice	OL-AT1, OL-AT2, OL-AT3, OL-AT4, OL-AT5	5	[23]
	Subjective norm on OL (SN OL)	Associated with support system of the PSMTs environment in using online learning mode during teaching practice	OL-SN1, OL-SN2, OL-SN3, OL-SN4	4	[23]
	Perceive of Behavioral Control on OL (PBC OL)	Associated with internal and external enablers/constraints in using online learning mode during teaching practice	OL-PBC1, OL-PBC2, OL-PBC3, OL-PBC4, OL-PBC5	5	[23]
The Use of DMLM	UDMLM	UDMLM during the online teaching practice reflected on their evaluation	UDMLM1, UDMLM2, UDMLM3, UDMLM4, UDMLM5, UDMLM6	6	[15]

Total				63

**Table 5:** Mean, SD, kurtosis, and skewness.

Components	N	Mean	Std. Deviation	Skewness		Kurtosis	
	Statistic	Statistic	Statistic	Statistic	Std. Error	Statistic	Std. Error
TK1	303	2.6304	0.69196	−0.084	0.140	−0.180	0.279
TK2	303	2.5644	0.66734	0.032	0.140	−0.222	0.279
TK3	303	2.7690	0.59197	−0.368	0.140	0.438	0.279
CK1	303	2.6271	0.66824	−0.206	0.140	−0.063	0.279
CK2	303	2.5908	0.58472	−0.208	0.140	−0.333	0.279
CK3	303	2.5578	0.64268	0.273	0.140	−0.329	0.279
CK4	303	2.4158	0.61333	0.065	0.140	−0.271	0.279
PK1	303	2.6997	0.67517	−0.268	0.140	0.063	0.279
PK2	303	2.5512	0.61697	−0.366	0.140	−0.176	0.279
PK3	303	2.7261	0.73301	−0.029	0.140	−0.099	0.279
PK4	303	2.5380	0.68887	0.230	0.140	−0.266	0.279
PK5	303	2.5974	0.76096	−0.628	0.140	−0.046	0.279
PK6	303	2.5116	0.74075	−0.458	0.140	1.350	0.279
PCK1	303	2.4455	0.57788	0.162	0.140	−0.480	0.279
PCK2	303	2.6337	0.60444	−0.254	0.140	−0.094	0.279
PCK3	303	2.5908	0.57903	−0.359	0.140	−0.280	0.279
PCK4	303	2.6634	0.56867	−0.722	0.140	0.332	0.279
PCK5	303	2.5941	0.54308	0.119	0.140	−1.010	0.279
TCK1	303	2.5941	0.60645	−0.680	0.140	0.095	0.279
TCK2	303	2.6238	0.67380	−0.295	0.140	−0.004	0.279
TCK3	303	2.5479	0.60641	−0.454	0.140	−0.202	0.279
TCK4	303	2.5578	0.66795	−0.414	0.140	−0.064	0.279
TPK1	303	2.5809	0.63499	0.162	0.140	−0.326	0.279
TPK2	303	2.6502	0.59501	−0.361	0.140	0.016	0.279
TPK3	303	2.5644	0.67229	−0.467	0.140	−0.029	0.279
TPK4	303	2.4290	0.69584	−0.753	0.140	1.536	0.279
MTPACK1	303	2.6403	0.64023	−0.575	0.140	0.279	0.279
MTPACK2	303	2.6436	0.68933	−0.253	0.140	−0.033	0.279
MTPACK3	303	2.5875	0.74013	−0.452	0.140	−0.097	0.279
MTPACK4	303	2.7261	0.67172	−0.537	0.140	0.462	0.279
MTPACK5	303	2.6568	0.72856	−0.402	0.140	0.020	0.279
DMLM-AT1	303	2.6238	0.69318	0.299	0.140	−0.459	0.279
DMLM-AT2	303	2.7558	0.61957	−0.289	0.140	0.254	0.279
DMLM-AT3	303	2.9472	0.63859	−0.415	0.140	0.770	0.279
DMLM-AT4	303	2.9868	0.57528	−0.001	0.140	0.051	0.279
DMLM-AT5	303	2.9736	0.59186	−0.573	0.140	1.754	0.279
DMLM-SN1	303	2.6634	0.52634	−0.136	0.140	−0.864	0.279
DMLM-SN2	303	2.9637	0.56557	−0.009	0.140	0.145	0.279
DMLM-SN3	303	3.0594	0.62224	−0.040	0.140	−0.412	0.279
DMLM-SN4	303	2.6733	0.55984	−0.715	0.140	0.330	0.279
DMLM-PCB1	303	2.9736	0.56319	−0.008	0.140	0.178	0.279
DMLM-PCB2	303	2.6502	0.63797	−0.154	0.140	−0.086	0.279
DMLM-PCB3	303	2.5974	0.55457	0.078	0.140	−0.813	0.279
DMLM-PCB4	303	2.5314	0.67953	0.013	0.140	−0.211	0.279
OL-AT1	303	1.9109	1.14307	−0.012	0.140	−0.861	0.279
OL-AT2	303	1.9274	1.03002	0.091	0.140	−0.736	0.279
OL-AT3	303	1.9703	1.00451	−0.098	0.140	−0.761	0.279
OL-AT4	303	2.0297	1.03695	0.012	0.140	−0.615	0.279
OL-AT5	303	1.9769	1.01126	−0.012	0.140	−0.680	0.279
OL-SN1	303	2.2013	0.80297	0.238	0.140	−0.419	0.279
OL-SN2	303	2.4917	0.69941	−0.321	0.140	−0.238	0.279
OL-SN3	303	2.4983	0.71351	0.143	0.140	−0.241	0.279
OL-SN4	303	2.3729	0.84739	−0.435	0.140	0.026	0.279
OL-PCB1	303	1.8746	0.95812	0.072	0.140	−0.524	0.279
OL-PCB2	303	2.2772	0.83925	−0.154	0.140	0.064	0.279
OL-PCB3	303	2.2904	0.78572	0.180	0.140	−0.361	0.279
OL-PCB4	303	2.7888	0.84274	−0.352	0.140	−0.401	0.279
OL-PCB5	303	2.7756	0.88912	−0.198	0.140	−0.763	0.279
UDMLM1	303	2.5644	0.68207	−0.392	0.140	−0.064	0.279
UDMLM2	303	2.5545	0.67316	−0.364	0.140	−0.090	0.279
UDMLM3	303	2.5545	0.67807	−0.071	0.140	−0.187	0.279
UDMLM4	303	2.6073	0.66677	−0.230	0.140	−0.067	0.279
UDMLM5	303	2.4983	0.67537	−0.221	0.140	−0.208	0.279
UDMLM6	303	2.4917	0.65541	−0.360	0.140	−0.232	0.279

Valid N (listwise)	303

**Table 6:** Reflective indicator loadings, internal consistency, composite reliability, and convergent validity.

Constructs	Items	Load	Cronbachʼs Alpha	Composite reliability	AVE
**CK**	CK1	0.734	0.843	0.893	0.677
	CK2	0.777			
	CK3	0.805			
	CK4	0.759			
**M-TPACK**	M-TPACK1	0.877	0.940	0.954	0.806
	M-TPACK2	0.863			
	M-TPACK3	0.810			
	M-TPACK4	0.912			
	M-TPACK5	0.826			
**PCK**	PCK1	0.728	0.923	0.942	0.766
	PCK2	0.868			
	PCK3	0.864			
	PCK4	0.796			
	PCK5	0.840			
**PK**	PK1	0.879	0.864	0.898	0.596
	PK2	0.823			
	PK3	0.883			
	PK4	0.893			
	PK5	0.881			
	PK6	0.873			
**TCK**	TCK1	0.740	0.876	0.915	0.729
	TCK2	0.712			
	TCK3	0.756			
	TCK4	**0.630**			
**TK**	TK1	0.844	0.716	0.841	0.638
	TK2	0.836			
	TK3	0.843			
**TPK**	TPK1	0.911	0.804	0.872	0.631
	TPK2	0.916			
	TPK3	0.923			
	TPK4	0.908			
**DMLM-AT**	DMLM-AT1	0.778	0.895	0.922	0.703
	DMLM-AT2	0.830			
	DMLM-AT3	0.816			
	DMLM-AT4	0.778			
	DMLM-AT5	0.767			
**DMLM-PBC**	DMLM-PCB1	**0.503**	0.771	0.867	0.685
	DMLM-PCB2	**0.717**			
	DMLM-PCB3	0.827			
	DMLM-PCB4	0.814			
**DMLM-SN**	DMLM-SN1	**0.407**	0.785	0.857	0.600
	DMLM-SN2	0.874			
	DMLM-SN3	0.764			
	DMLM-SN4	**0.677**			
**OL-AT**	OL-AT1	0.903	0.958	0.967	0.855
	OL-AT2	0.877			
	OL-AT3	0.857			
	OL-AT4	0.806			
	OL-AT5	0.772			
**OL-PBC**	OL-PCB1	0.808	0.876	0.910	0.669
	OL-PCB2	0.817			
	OL-PCB3	0.728			
	OL-PCB4	0.863			
	OL-PCB5	0.736			
**OL-SN**	OL-SN1	**0.659**	0.917	0.942	0.802
	OL-SN2	0.826			
	OL-SN3	0.832			
	OL-SN4	0.794

## 4 Experimental Design, Materials and Methods

In this study, the scale was developed in two stages. The first phase involves adapting and translating the research instrument. The adaptation process began with a systematic literature review of the instruments used by previous researchers to measure the variables of TPACK, TPB, and the use of DMLM. The findings of the literature review were then modified considering the research objectives (see Table 3 for a general overview of the instrument). Furthermore, the developed instruments are transliterated from Indonesian to English and vice versa by two linguists from one of Indonesiaʼs universities. The second phase involves face and content validity, which is accomplished through two discussions. The first meeting was held. The next face-to-face discussion involved five PSMTs to ensure that the instruments developed were easy to use and understand. Passing the 2-phase procedures, the instrument then was administered online by using Google Form to collect survey data starting from October to November 2022 through simple random sampling. The instrument was distributed online via Google Form to preservice mathematics teachers from 14 mathematics education departments across 11 Indonesian provinces, yielding 303 responses for further analysis.

The data collected is then organized into Microsoft Excel. The data was then tested for normality by calculating the level of Skewness and Kurtosis by using SPSS 23 with a normality category of <±1.96 [24]. Table 5 shows that all items' Skewness and Kurtosis values are in the threshold range: Skewness ranges from −0.758 (TPK4) to 0.299 (DMLM-AT1) with a standard deviation of 0.140, and Kurtosis ranges from −1.010 (PCK5) to 1.754 (DMLM-AT5) with a standard deviation of 0.279. The following analysis is related to the four measurement model assessments, reflective indicator loadings, internal consistency reliability, discriminant, and convergent validity—performed in SmartPLS 3 (see Table 6). The loadings of reflective indicator should be equal or higher than 0.708 [15]. Table 5 shows the values of the reflective loading indicator from 58 items tested from 14 constructs. From the table, there are five items that have values below 0.708 (read color) which are TCK4 (0.630), DMLM-PCB1 (0.503), DMLM-SN1 (0.407), DMLM-SN4 (0.677), and OL-SN1 (0.659). Therefore, the five items are then dropped, then retested using SmartPLS 3 and obtained cross loading results that have been cleaned from the five items as shown in Table 8.

In Table 6, the internal consistency should be measured using Cronbach's alpha and a Composite Reliability (CR) higher than.700 [3,11]. The datasets gain Cronbach's alpha score ranging from 0.771 to 0.958, and CR score from 0.841 to 0.954. Therefore, all constructs prove reliable. Therefore, all constructions prove are reliable. The validity of convergent was reported through Average Variance Extracted (AVE) where the minimum value of 0.500 should be gained [26]. Table 6 shows that the AVE values from the dataset range from 0.596 to 0.855. The Fornell-other Larcker's construct was used to assess discriminant validity [27]. The data revealed that the values of each construct are smaller than their combined variance (Table 7). When the loading on a construct is larger than the loading on other constructs, the discriminant validity is provided; cross-loading values.

**Table 7:** Fornell–Larcker criterion.

Construct	CK	DMLM-SN	DMLM-AT	DMLM-PBC	M-TPACK	OL-AT	OL-PBC	OL-SN	PCK	PK	TCK	TK	TPK	UDMLM
**CK**	0.824													
**DMLM-SN**	0.189	0.932												
**DMLM-AT**	0.127	0.493	0.838											
**DMLM-PBC**	0.133	0.327	0.393	0.827										
**M-TPACK**	0.206	0.117	0.205	0.358	0.898									
**OL-AT**	0.196	0.063	0.274	0.202	0.203	0.925								
**OL-PBC**	0.154	0.070	0.281	0.314	0.388	0.655	0.818							
**OL-SN**	0.167	0.152	0.340	0.184	0.264	0.679	0.722	0.927						
**PCK**	0.380	0.424	0.343	0.224	0.396	0.210	0.315	0.391	0.875					
**PK**	0.380	0.351	0.211	0.158	0.498	0.267	0.286	0.394	0.703	0.772				
**TCK**	0.348	0.263	0.464	0.400	0.517	0.111	0.260	0.261	0.434	0.405	0.888			
**TK**	0.482	0.206	0.317	0.197	0.277	−0.036	0.094	0.225	0.433	0.372	0.599	0.799		
**TPK**	0.335	0.357	0.356	0.333	0.640	0.228	0.296	0.384	0.614	0.661	0.641	0.471	0.794	
**UDMLM**	0.068	0.186	0.516	0.451	0.543	0.427	0.625	0.585	0.449	0.414	0.445	0.300	0.558	0.860

**Table 8:** Cross loading.

Constructs	CK	DMLM-SN	DMLM_AT	DMLM_PBC	M-TPACK	OL_AT	OL_PBC	OL_SN	PCK	PK	TCK	TK	TPK	UDMLM
**CK1**	**0.770**	0.049	0.064	0.055	−0.038	−0.005	0.060	0.066	0.093	0.201	0.371	0.491	0.176	−0.060
**CK2**	**0.849**	0.288	0.180	0.132	0.150	0.189	0.077	0.101	0.393	0.273	0.218	0.446	0.222	0.045
**CK3**	**0.873**	0.161	0.153	0.079	0.148	0.216	0.111	0.194	0.371	0.344	0.285	0.365	0.263	0.065
**CK4**	**0.799**	0.100	0.010	0.158	0.363	0.198	0.242	0.168	0.337	0.405	0.301	0.324	0.417	0.140
**DMLM-AT1**	0.074	0.208	**0.795**	0.420	0.230	0.232	0.248	0.270	0.174	0.162	0.389	0.317	0.240	0.404
**DMLM-AT2**	0.154	0.353	**0.890**	0.383	0.301	0.180	0.303	0.254	0.383	0.235	0.527	0.302	0.406	0.591
**DMLM-AT3**	0.059	0.490	**0.855**	0.268	0.069	0.381	0.263	0.387	0.235	0.111	0.258	0.210	0.264	0.386
**DMLM-AT4**	0.115	0.514	**0.812**	0.359	0.068	0.128	0.141	0.145	0.256	0.169	0.430	0.260	0.288	0.334
**DMLM-AT5**	0.110	0.583	**0.835**	0.186	0.106	0.247	0.176	0.385	0.355	0.183	0.280	0.224	0.242	0.360
**DMLM-PCB2**	0.165	0.413	0.404	**0.779**	0.160	0.024	0.104	0.038	0.176	0.143	0.489	0.298	0.258	0.292
**DMLM-PCB3**	0.055	0.342	0.295	**0.888**	0.368	0.207	0.280	0.181	0.225	0.142	0.383	0.177	0.406	0.421
**DMLM-PCB4**	0.129	0.090	0.303	**0.812**	0.326	0.234	0.360	0.209	0.153	0.111	0.161	0.048	0.150	0.388
**DMLM-SN2**	0.171	**0.914**	0.432	0.257	0.041	0.011	−0.002	0.163	0.350	0.247	0.214	0.199	0.246	0.150
**DMLM-SN3**	0.181	**0.949**	0.483	0.343	0.162	0.096	0.119	0.126	0.431	0.391	0.270	0.187	0.401	0.193
**M-TPACK1**	0.200	0.086	0.177	0.355	**0.926**	0.171	0.386	0.274	0.379	0.480	0.483	0.267	0.589	0.518
**M-TPACK2**	0.107	0.121	0.130	0.303	**0.860**	0.238	0.386	0.189	0.345	0.439	0.320	0.254	0.558	0.467
**M-TPACK3**	0.189	0.037	0.253	0.354	**0.866**	0.224	0.352	0.280	0.347	0.402	0.548	0.278	0.556	0.466
**M-TPACK4**	0.181	0.172	0.179	0.244	**0.935**	0.126	0.328	0.180	0.303	0.433	0.454	0.255	0.620	0.492
**M-TPACK5**	0.246	0.108	0.180	0.351	**0.901**	0.158	0.294	0.259	0.404	0.483	0.510	0.191	0.547	0.493
**OL-AT1**	0.142	0.056	0.264	0.154	0.159	**0.941**	0.576	0.580	0.220	0.209	0.001	−0.102	0.166	0.349
**OL-AT2**	0.149	−0.030	0.177	0.217	0.130	**0.923**	0.652	0.587	0.124	0.163	0.068	−0.088	0.172	0.368
**OL-AT3**	0.248	0.016	0.233	0.242	0.183	**0.916**	0.606	0.597	0.150	0.272	0.227	0.006	0.239	0.366
**OL-AT4**	0.192	0.140	0.311	0.135	0.227	**0.916**	0.533	0.630	0.246	0.287	0.151	−0.019	0.248	0.427
**OL-AT5**	0.172	0.089	0.268	0.191	0.223	**0.928**	0.660	0.722	0.220	0.289	0.061	0.018	0.218	0.445
**OL-PCB1**	−0.033	−0.154	0.074	0.328	0.298	0.654	**0.757**	0.590	0.209	0.236	0.092	−0.035	0.175	0.466
**OL-PCB2**	0.078	−0.091	0.102	0.237	0.300	0.564	**0.845**	0.508	0.191	0.221	0.161	0.016	0.186	0.463
**OL-PCB3**	0.066	0.067	0.304	0.396	0.301	0.631	**0.859**	0.580	0.277	0.261	0.167	−0.013	0.245	0.567
**OL-PCB4**	0.253	0.156	0.333	0.132	0.368	0.377	**0.824**	0.593	0.279	0.224	0.309	0.181	0.293	0.534
**OL-PCB5**	0.249	0.273	0.298	0.188	0.317	0.466	**0.801**	0.679	0.319	0.225	0.321	0.225	0.301	0.510
**OL-SN2**	0.082	0.180	0.344	0.235	0.276	0.645	0.740	**0.948**	0.282	0.302	0.284	0.227	0.308	0.594
**OL-SN3**	0.231	0.126	0.321	0.134	0.285	0.543	0.608	**0.909**	0.477	0.421	0.340	0.284	0.514	0.553
**OL-SN4**	0.159	0.110	0.272	0.132	0.156	0.714	0.654	**0.924**	0.332	0.379	0.073	0.097	0.230	0.464
**PCK1**	0.331	0.342	0.202	0.162	0.325	0.153	0.196	0.289	**0.827**	0.640	0.454	0.470	0.569	0.306
**PCK2**	0.355	0.446	0.347	0.186	0.318	0.113	0.206	0.295	**0.910**	0.628	0.405	0.379	0.501	0.349
**PCK3**	0.307	0.287	0.260	0.190	0.384	0.135	0.263	0.301	**0.926**	0.648	0.378	0.386	0.620	0.385
**PCK4**	0.366	0.383	0.380	0.256	0.433	0.298	0.435	0.461	**0.870**	0.655	0.380	0.337	0.575	0.554
**PCK5**	0.296	0.413	0.312	0.179	0.234	0.225	0.261	0.364	**0.837**	0.468	0.251	0.312	0.377	0.345
**PK1**	0.314	0.283	0.088	0.143	0.302	0.050	0.078	0.209	0.507	**0.720**	0.390	0.500	0.516	0.164
**PK2**	0.349	0.352	0.146	0.200	0.321	0.119	0.201	0.333	0.567	**0.714**	0.339	0.313	0.408	0.168
**PK3**	0.291	0.207	0.135	0.054	0.312	0.225	0.193	0.249	0.530	**0.777**	0.265	0.203	0.503	0.263
**PK4**	0.268	0.256	0.020	0.074	0.383	0.175	0.222	0.259	0.590	**0.787**	0.182	0.258	0.461	0.343
**PK5**	0.206	0.225	0.175	0.063	0.443	0.274	0.266	0.321	0.467	**0.825**	0.266	0.149	0.492	0.380
**PK6**	0.325	0.300	0.376	0.187	0.520	0.364	0.342	0.430	0.581	**0.802**	0.417	0.293	0.650	0.551
**TCK1**	0.226	0.208	0.412	0.331	0.470	0.015	0.223	0.193	0.336	0.262	**0.895**	0.543	0.506	0.368
**TCK2**	0.327	0.231	0.382	0.388	0.433	0.084	0.174	0.156	0.366	0.407	**0.912**	0.609	0.572	0.356
**TCK3**	0.380	0.264	0.448	0.346	0.479	0.206	0.304	0.361	0.461	0.414	**0.855**	0.434	0.635	0.469
**TK1**	0.346	0.112	0.297	0.160	0.120	−0.056	0.086	0.298	0.333	0.246	0.430	**0.795**	0.385	0.306
**TK2**	0.334	0.263	0.225	0.173	0.246	−0.064	0.019	0.106	0.332	0.244	0.439	**0.747**	0.330	0.161
**TK3**	0.462	0.131	0.243	0.143	0.289	0.021	0.111	0.142	0.371	0.385	0.556	**0.852**	0.410	0.250
**TPK1**	0.259	0.241	0.454	0.364	0.411	0.051	0.170	0.197	0.439	0.414	0.620	0.559	**0.730**	0.379
**TPK2**	0.310	0.401	0.397	0.224	0.510	0.132	0.174	0.245	0.540	0.532	0.668	0.387	**0.836**	0.373
**TPK3**	0.253	0.242	0.267	0.247	0.505	0.276	0.317	0.447	0.385	0.507	0.492	0.261	**0.829**	0.538
**TPK4**	0.244	0.246	0.055	0.234	0.584	0.249	0.274	0.326	0.564	0.621	0.289	0.309	**0.777**	0.477
**UDMLM1**	0.173	0.190	0.457	0.489	0.508	0.454	0.524	0.649	0.420	0.404	0.432	0.299	0.507	**0.827**
**UDMLM2**	−0.021	0.098	0.360	0.438	0.511	0.418	0.530	0.489	0.270	0.328	0.317	0.225	0.479	**0.869**
**UDMLM3**	0.132	0.032	0.484	0.364	0.582	0.399	0.612	0.537	0.502	0.470	0.450	0.279	0.606	**0.869**
**UDMLM4**	0.025	0.252	0.466	0.454	0.551	0.314	0.475	0.384	0.422	0.366	0.326	0.200	0.501	**0.910**
**UDMLM5**	0.019	0.229	0.470	0.295	0.292	0.287	0.521	0.443	0.393	0.246	0.396	0.215	0.384	**0.847**
**UDMLM6**	−0.018	0.193	0.418	0.249	0.289	0.296	0.550	0.483	0.272	0.275	0.356	0.326	0.351	**0.837**

## Limitations

The limitation in this dataset is on data collection locations that focus in Indonesia and are limited to only 14 universities in 11 provinces. Furthermore, this dataset is the result of a pilot study used to test the validity and reliability of the questionnaire developed. In the aspect of data analysis techniques, the software used in this study only focuses on SPSS 23 and SmartPLS 3. In the aspect of survey respondents, 73 % of them are women which makes this condition a limitation in this study. This condition is closely related to field conditions where most pre-service mathematics teachers in the 14 universities where the data is collected are female. However, this data is intended to verify the level of validity and reliability of instruments developed through the Exploratory Factor Analysis technique, not in real studies that must really pay attention to gender bias where demographical background is essential to be considered. Therefore, the data is still useful to be used and analysed.

## Ethics Statement

For the data collection, informed consent was acquired, and participation was entirely voluntary. The survey was anonymous, and no personal information about the participants was collected. The instrument has passed the research ethic by University Malaya Research Ethic Committee (UMREC) with letter number **UM.TNC2/UMREC_2217**.

## CRediT Author Statement

**Naufal Ishartono:** Data curation, Conceptualization, Methodology, Writing original draft preparation, Software **Siti Hajar binti Halili:** Visualization, Supervision **Rafiza binti Abdul Razak:** Supervision, Validation, Writing-Reviewing and Editing.

## Data Availability

Mendeley DataPilot Data of Instrument Validation for Factor Affecting Indonesian Preservice Mathematics Teachers' Technology Integration (Original data).
